# Supplementary material for: The Small RNA Universe of Capitella teleta
Source: Front Mol Biosci. 2022 Feb 25;9:802814. doi: 10.3389/fmolb.2022.802814 (PMC8915122; doi:10.3389/fmolb.2022.802814)
Supplement: Supplementary file 1 [file DataSheet1.ZIP › Supplement/known/CAPTEscaffold_107_7117.pdf]

Provisional ID	:	CAPTEscaffold_107_7117
Score total	:	6492.2
Score for star read(s)	:	3.9
Score for read counts	:	6482.6
Score for mfe	:	1.2
Score for randfold	:	1.6
Score for cons. seed	:	3
Total read count	:	12727
Mature read count	:	7414
Loop read count	:	0
Star read count	:	5313

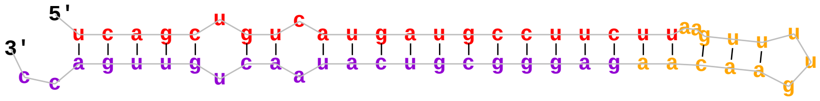

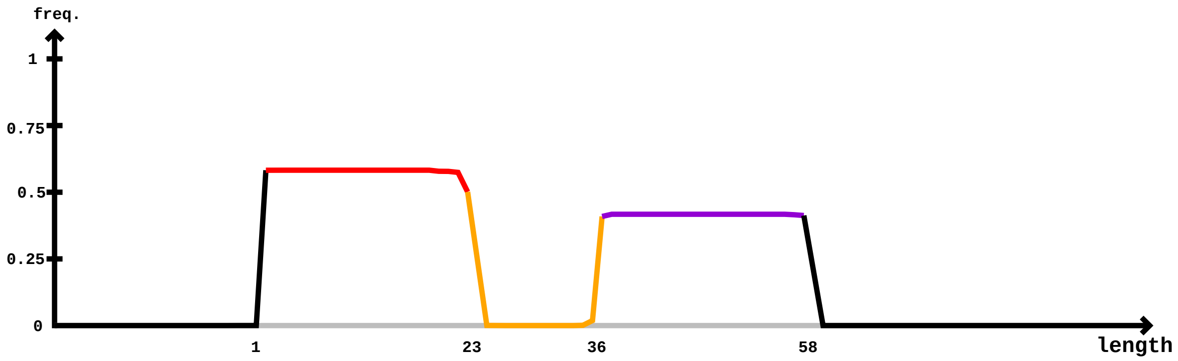


```
                    Mature                           Star
5'- uagaaaccaucccgaccaguucagcugucaugaugccuucuuaaguuuugaacaagagggcgucauaacuguugaccucgaagggaaccuuaccucaucugcuucgcucgcc   -3'   obs
    uagaaaccaucccgaccaguucagcugucaugaugccuucuuaaguuuugaacaagagggcgucauaacuguugaccucgaagggaaccuuaccucaucugcuucgcucgcc         exp
    uagaaaccaucccgaccaguucagcugucaugaugccuucuuaaguuuugaacaagagggcgucauaacuguugaccucgaagggaaccuuaccucaucugcuucgcucgcc         known
    .........((((..(.((.(((((.((.(((((((((((((..(((...)))))))))))))))).)).))))).)).)..)))).........................   reads   mm   sample

    .................aguucagcugucaugaugccu..........................................................................   1   0   seq
    ....................ucagcugucaugaugccu..........................................................................   48   0   seq
    ....................ucagcugucaugaugccA..........................................................................   1   1   seq
    ....................ucagcugucaugaugccG..........................................................................   1   1   seq
    ....................ucagcugucaugaugccuu.........................................................................   5   0   seq
    ....................ucagcugucaugaugccuuc........................................................................   49   0   seq
    ....................Gcagcugucaugaugccuucu.......................................................................   1   1   seq
    ....................ucagcugucaugaugccuuAu.......................................................................   1   1   seq
    ....................ucagcugucaugaugccuuUu.......................................................................   1   1   seq
    ....................ucagcugucaugaugccGucu.......................................................................   1   1   seq
    ....................ucagcuguUaugaugccuucu.......................................................................   2   1   seq
    ....................ucagcugucaugaugUcuucu.......................................................................   1   1   seq
    ....................ucagcugAcaugaugccuucu.......................................................................   2   1   seq
    ....................ucagcugucaugaAgccuucu.......................................................................   1   1   seq
    ....................Acagcugucaugaugccuucu.......................................................................   3   1   seq
    ....................ucagcugucaugaugccuucG.......................................................................   1   1   seq
    ....................ucagcugucauAaugccuucu.......................................................................   1   1   seq
    ....................ucagcugucaugaugccuucu.......................................................................   908   0   seq
    ....................ucagcugucaugaugccuucA.......................................................................   3   1   seq
    ....................ucagcAgucaugaugccuucu.......................................................................   2   1   seq
    ....................ucagcugucaugauUccuucu.......................................................................   1   1   seq
    ....................ucagcugucaugaAgccuucuu......................................................................   2   1   seq
    ....................ucagcugucaugaCgccuucuu......................................................................   2   1   seq
    ....................ucagcugucaugaugcAuucuu......................................................................   1   1   seq
    ....................Gcagcugucaugaugccuucuu......................................................................   4   1   seq
    ....................ucagcugGcaugaugccuucuu......................................................................   1   1   seq
    ....................uAagcugucaugaugccuucuu......................................................................   1   1   seq
    ....................ucagcuguAaugaugccuucuu......................................................................   1   1   seq
    ....................ucagcugucaugaugccAucuu......................................................................   4   1   seq
    ....................ucaUcugucaugaugccuucuu......................................................................   1   1   seq
    ....................ucGgcugucaugaugccuucuu......................................................................   3   1   seq
```

```
                    Mature                             Star
uagaaaccaucccgaccaguucagcugucaugaugccuucuuaaguuuugaacaagagggcgucauaacuguugaccucgaagggaaccuuaccucaucugcuucgcucgcc
....................ucagcugucauAaugccuucuu......................................................................   14      1     seq
....................ucagUugucaugaugccuucuu......................................................................   1       1     seq
....................ucagcugucaugaugccuuGuu......................................................................   3       1     seq
....................Acagcugucaugaugccuucuu......................................................................   27      1     seq
....................ucagcugucaugGugccuucuu......................................................................   1       1     seq
....................ucagcugucaugaugccuucCu......................................................................   2       1     seq
....................Ncagcugucaugaugccuucuu......................................................................   2       1     seq
....................ucagcugucaugaugccuAcuu......................................................................   2       1     seq
....................ucagcuAucaugaugccuucuu......................................................................   1       1     seq
....................ucagcugAcaugaugccuucuu......................................................................   2       1     seq
....................ucagcugucaugaugccuucGu......................................................................   1       1     seq
....................ucagcugucaugaugccuCcuu......................................................................   1       1     seq
....................ucagcugucaugaugcUuucuu......................................................................   2       1     seq
....................ucagcugucaAgaugccuucuu......................................................................   4       1     seq
....................ucagcugucauCaugccuucuu......................................................................   1       1     seq
....................ucagGugucaugaugccuucuu......................................................................   1       1     seq
....................ucagcAgucaugaugccuucuu......................................................................   1       1     seq
....................ucagcugucaugaugccuucuu......................................................................   6121    0     seq
....................ucagcCgucaugaugccuucuu......................................................................   3       1     seq
....................ucagcuguUaugaugccuucuu......................................................................   2       1     seq
....................ucagcugucaugaugUcuucuu......................................................................   1       1     seq
....................ucagcugucaugaugccuucuA......................................................................   4       1     seq
....................uUagcugucaugaugccuucuu......................................................................   6       1     seq
....................ucagcugucauUaugccuucuu......................................................................   2       1     seq
....................ucagAugucaugaugccuucuu......................................................................   1       1     seq
....................ucagcugucaugaugccuuAuu......................................................................   3       1     seq
....................ucagcugucaugaugccuucuuU.....................................................................   94      1     seq
....................ucagcugucaugaugccuucuua.....................................................................   51      0     seq
....................ucagcugucaugaugccuucuuaa....................................................................   3       0     seq
....................ucagcugucaugaugccuucuuaaU...................................................................   1       1     seq
....................ucagcugucaugaugccuucuuaaA...................................................................   1       1     seq
.....................cagcugucaugaugccuucuu......................................................................   1       0     seq
......................agcugucaugaugccuucuu......................................................................   1       0     seq
.....................................................aagagggcgucauaacuguuga.....................................   12      0     seq
......................................................agagggcgucauaacuguuga.....................................   7       0     seq
......................................................agagggcgucauaacuguugac....................................   19      0     seq
......................................................agaAggcgucauaacuguugacc...................................   1       1     seq
......................................................agagggcgucGuaacuguugacc...................................   1       1     seq
......................................................agagggcgucauaacuguugacU...................................   1       1     seq
......................................................agaggAgucauaacuguugacc...................................   1       1     seq
......................................................agagggcgucauaacuguugacc...................................   199     0     seq
.......................................................gagggcgucauaacuguuga.....................................   6       0     seq
.......................................................gagggcgucauaacuguugac....................................   16      0     seq
.......................................................gagggcguGauaacuguugacc...................................   1       1     seq
.......................................................gagggcgucUuaacuguugacc...................................   2       1     seq
.......................................................gagggcgAcauaacuguugacc...................................   3       1     seq
.......................................................gagggcgucauaacuguCgacc...................................   1       1     seq
.......................................................gagggUgucauaacuguugacc...................................   2       1     seq
.......................................................gagAgcgucauaacuguugacc...................................   3       1     seq
.......................................................gagggcgucauaacuguugaAc...................................   1       1     seq
.......................................................Uagggcgucauaacuguugacc...................................   9       1     seq
.......................................................gagggcgucauaacuCuugacc...................................   1       1     seq
.......................................................gagggcgucauaacugAugacc...................................   1       1     seq
.......................................................gagggcgucauaacuguugacc...................................   4815    0     seq
.......................................................gagggcgucauGacuguugacc...................................   1       1     seq
.......................................................gaAggcgucauaacuguugacc...................................   9       1     seq
.......................................................gagCgcgucauaacuguugacc...................................   1       1     seq
.......................................................gagggcgucauaacCguugacc...................................   1       1     seq
.......................................................gagggcgucauaacuguugacG...................................   1       1     seq
.......................................................Aagggcgucauaacuguugacc...................................   43      1     seq
.......................................................gagggcgucauaacuguugaGc...................................   1       1     seq
.......................................................gaggAcgucauaacuguugacc...................................   1       1     seq
.......................................................gagggcgucauaacuguuAacc...................................   4       1     seq
.......................................................gagggcgucauaacuguugCcc...................................   1       1     seq
.......................................................gagggcgucauaacGguugacc...................................   1       1     seq
.......................................................gagggcgucauaacuguugacU...................................   3       1     seq
.......................................................gGgggcgucauaacuguugacc...................................   15      1     seq
.......................................................gaggCcgucauaacuguugacc...................................   2       1     seq
.......................................................gagggcgucauaUcuguugacc...................................   1       1     seq
.......................................................gagggcAucauaacuguugacc...................................   4       1     seq
```

```
                    Mature                              Star
uagaaaccaucccgaccaguucagcugucaugaugccuucuuaaguuuuugaacaagagggcgucauaacuguugaccucgaagggaaccuuaccucaucugcuucgcucgcc
........................................................gagggcgucauaGcuguugacc...................................    2    1    seq
........................................................gagggcgucauaacuguAgacc...................................    2    1    seq
........................................................gagggcgucauaacuguugUcc...................................    1    1    seq
........................................................gaUggcgucauaacuguugacc...................................    1    1    seq
........................................................gagggcguAauaacuguugacc...................................    1    1    seq
........................................................gagggcgucauaacAguugacc...................................    2    1    seq
........................................................Cagggcgucauaacuguugacc...................................    1    1    seq
........................................................gagggcgucauaacuguugaccu..................................    6    0    seq
.........................................................aAggcgucauaacuguugacc...................................    1    1    seq
.........................................................Ggggcgucauaacuguugacc...................................    2    1    seq
.........................................................agggcgucaAaacuguugacc...................................    1    1    seq
.........................................................agggcgucauaacuguugacc...................................    102  0    seq
```
